# Neuron type-specific expression of a mutant KRAS impairs hippocampal-dependent learning and memory

**DOI:** 10.1038/s41598-020-74610-y

**Published:** 2020-10-20

**Authors:** Hyun-Hee Ryu, Minkyung Kang, Kyoung-Doo Hwang, Han Byul Jang, Sang Jeong Kim, Yong-Seok Lee

**Affiliations:** 1 Department of Physiology, Seoul National University College of Medicine, Seoul, 03080 Korea; 2 Neuroscience Research Institute, Seoul National University College of Medicine, Seoul, 03080 Korea; 3 BK21-Plus Biomedical Science Project, Seoul National University College of Medicine, Seoul, 03080 Korea; 4 Department of Biomedical Sciences, Seoul National University College of Medicine, Seoul, 03080 Korea

**Keywords:** Diseases of the nervous system, Learning and memory, Molecular neuroscience

## Abstract

*KRAS* mutations are associated with rare cases of neurodevelopmental disorders that can cause intellectual disabilities. Previous studies showed that mice expressing a mutant *KRAS* have impaired the development and function of GABAergic inhibitory neurons, which may contribute to behavioural deficits in the mutant mice. However, the underlying cellular mechanisms and the role of excitatory neurons in these behavioural deficits in adults are not fully understood. Herein, we report that neuron type-specific expression of a constitutively active mutant KRAS^G12V^ in either excitatory or inhibitory neurons resulted in spatial memory deficits in adult mice. In inhibitory neurons, KRAS^G12V^ induced ERK activation and enhanced GABAergic synaptic transmission. Expressing KRAS^G12V^ in inhibitory neurons also impaired long-term potentiation in the hippocampal Shaffer-collateral pathway, which could be rescued by picrotoxin treatment. In contrast, KRAS^G12V^ induced ERK activation and neuronal cell death in excitatory neurons, which might have contributed to the severe behavioural deficits. Our results showed that both excitatory and inhibitory neurons are involved in mutant *KRAS*-associated learning deficits in adults via distinct cellular mechanisms.

## Introduction

The RAS-extracellular signal-regulated kinase (ERK) signalling pathway is involved in diverse cellular processes including cell proliferation, differentiation, and growth^1,2^. Mutations in genes encoding the RAS-ERK signalling pathway proteins are associated with multiple genetic disorders, collectively termed RASopathies. These include Noonan syndrome (NS), neurofibromatosis, Costello syndrome, cardiofaciocutaneous syndrome (CFCS), LEOPARD syndrome, and others^3–6^. Common features of RASopathies are growth delay, congenital heart defects, and short stature^3–6^. Patients with RASopathies also show a wide range of cognitive deficits from mild learning defects to severe intellectual disabilities^4,7,8^.

RAS proteins which are small GTPases, acting as molecular switches of RAS-ERK signalling, include HRAS, KRAS, and NRAS^9,10^. To date, 52 *KRAS* mutations have been identified in NS and 19 have been identified in CFCS (NSEuroNet database, https://nseuronet.com). RASopathy-associated *KRAS* mutations are gain-of-function mutations, but most of these mutant proteins have a milder activity than those with oncogenic mutations^11,12^. Point mutations in codon 12 of the *KRAS* gene, such as G12V, account for approximately 80% of KRAS mutations in cancer^13,14^. However, G12S mutations are also reported in rare cases of RASopathies^15–17^. Mice expressing a constitutively active mutant KRAS^G12V^ in neurons under the control of synapsin promoter (Syn-cre;KRAS^G12V^) showed deficits in LTP as well as in spatial memory in adults^18^. GABAergic synaptogenesis was found to be significantly increased in Syn-cre;KRAS^G12V^ mice, suggesting that KRAS is involved in synaptogenesis during early development, dysregulation of which subsequently impairs synaptic plasticity and memory in adults^18^. Importantly, KRAS is expressed in the adult mouse brain as well^19^. However, the physiological functions of KRAS in adult neurons remain unclear. Furthermore, the cellular mechanisms underlying the mutant KRAS-associated deficits in synaptic plasticity, learning and memory in adult remain unknown.

In this study, we investigated the cell type-dependent roles of KRAS by expressing the mutant KRAS^G12V^ either in excitatory or in inhibitory neurons in adult mice. Expression of the mutant KRAS either in excitatory or in inhibitory neurons impaired learning and memory. We found that the mutant KRAS results in distinct cellular abnormalities depending on which neuronal cell types it is expressed in. The mutant KRAS increases synaptic transmission in inhibitory neurons, while it promotes the cell death of excitatory neurons. Our findings would contribute to understanding the physiological roles of KRAS as well as the pathophysiology of KRAS-associated RASopathies.

## Results

### Ectopic expression of KRAS^G12V^ in inhibitory neurons impairs spatial learning and memory

**Figure 1:**
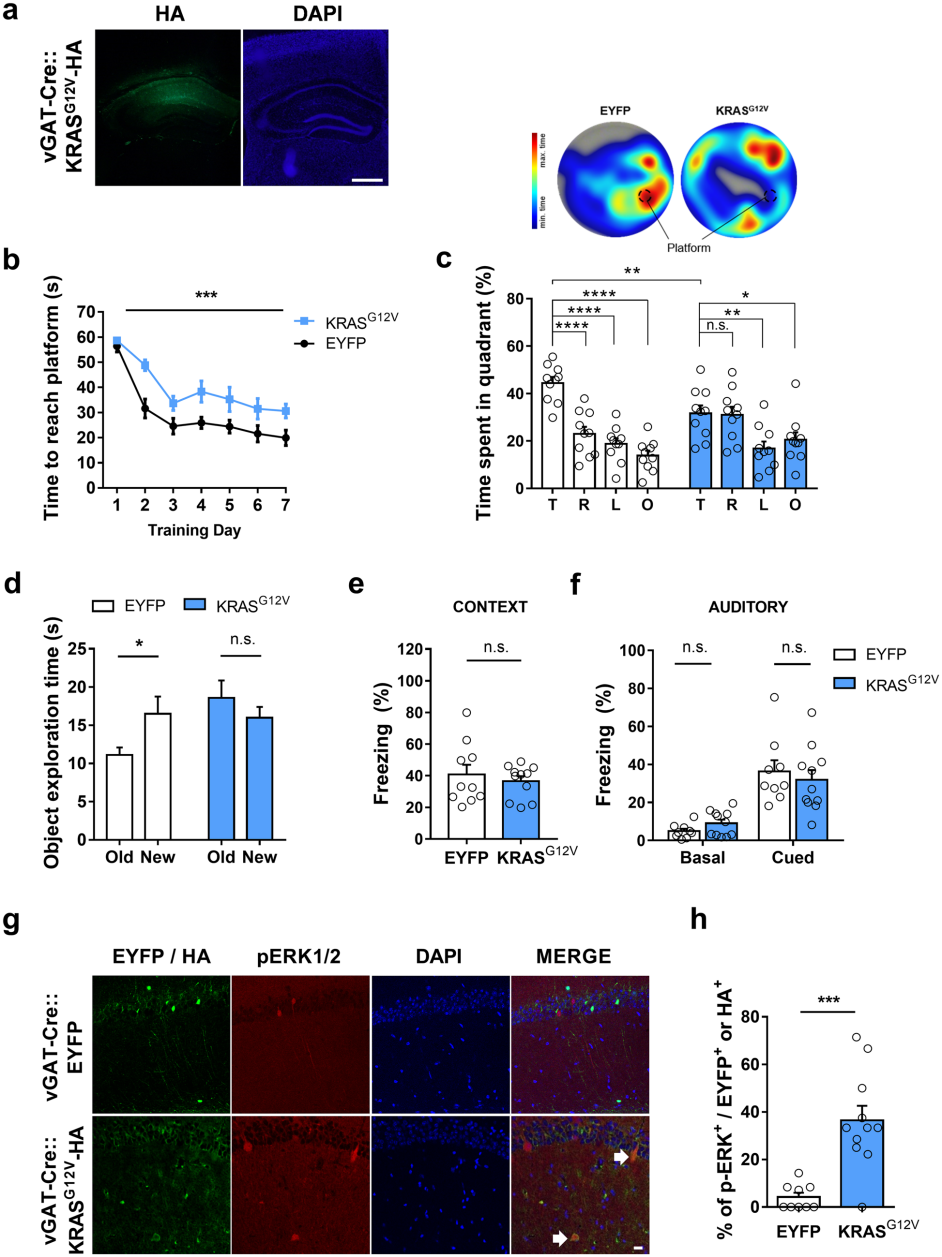
Ectopic expression of KRAS^G12V^ in inhibitory neurons causes spatial memory deficits and ERK hyperactivation. (**a**) Expression of adeno-associated virus (AAV) expressing KRAS^G12V^-HA in the dorsal CA regions of adult vGAT-Cre mouse. 4′,6-diamidino-2-phenylindole (DAPI) staining was used to identify nuclei. Scale bars, 200 μm. (**b**) Learning curve showing that the latency to find the hidden platform during the Morris water maze (MWM) training trials was significantly increased in vGAT-Cre::KRAS^G12V^ mice as compared to vGAT-Cre::EYFP. vGAT-Cre::EYFP, n = 10; vGAT-Cre::KRAS^G12V^, n = 10; Two-way repeated measures ANOVA, *F*_1, 18_ = 21.53, ***P = 0.0002. (**c**) Lower panel, time spent in each quadrant during the probe test. vGAT-CRE:: KRAS^G12V^ mice spent significantly less time in the target quadrant compared to vGAT-Cre::EYFP mice (comparison of target quadrant occupancy, unpaired t-test, **P = 0.0063). Moreover, vGAT-Cre::EYFP mice selectively searched for the platform in the target quadrant, while KRAS^G12V^ did not (vGAT-Cre::EYFP, n = 10; One-Way ANOVA, followed by Dunnett’s post hoc test, ****P < 0.0001; vGAT-Cre::KRAS^G12V^, n = 10; one-way ANOVA, followed by Dunnett’s post hoc test, *P* = 0.9982, **P = 0.0069, *P = 0.0493). T, target quadrant, R, right to target, L, left to target, O, opposite to target. Upper panel, representative heat map summary of MWM probe test. Platform position during the training trials is indicated by the dotted circle. (**d**) In object-place recognition test, vGAT-Cre::EYFP mice showed significant preference to the relocated (new) object, while vGAT-Cre::KRAS^G12V^ mice showed similar preference between old and new object. vGAT-Cre::EYFP, n = 26; paired t-test, *P = 0.0112; vGAT-Cre::KRAS^G12V^, n = 27; paired t-test, *P* = 0.1223; New, new location; old, object location used in training session. (**e**, **f**) In contextual (**e**) and cued (**f**) fear memory test, two groups showed comparable levels of freezing. Contextual test, vGAT-Cre::EYFP, n = 10, vGAT-Cre::KRAS^G12V^, n = 11, unpaired t-test, *P* = 0.534; Cued test, vGAT-Cre::EYFP, n = 9; vGAT-Cre::KRAS^G12V^, n = 11, unpaired t-test, *P* = 0.1157 for basal freezing before tone presented, *P* = 0.5690 for freezing in response to tone. (**g**) Representative image of HA and pErk staining of EYFP or KRAS^G12V^expressing hippocampal slices from vGAT-Cre mice. Slices were immunostained for p-ERK1/2 (red), HA (green), and DAPI (blue). White arrows indicate pERK1/2 and HA-KRAS^G12V^double labelled cells, Scale bar = 20 μm. **(h**) Percentage of pERK-positive neurons was significantly higher in the KRAS^G12V^expressing hippocampal slices. vGAT-Cre::EYFP, n = 9 slices from 3 hippocampi, vGAT-Cre::KRAS^G12V^, n = 11 slices from 4 hippocampi. ***P = 0.0002. Data are expressed as the mean ± SEM.

Previously, we have reported that *Kras* expression is significantly higher in inhibitory neurons than in excitatory neurons in the adult mouse hippocampus^19^. To assess the effects of neuronal type-specific expression of KRAS^G12V^ on learning and synaptic plasticity in adult mice, we injected a Cre recombinase–dependent adeno-associated virus (AAV) vector encoding KRAS^G12V^–hemagglutinin (HA) into the dorsal hippocampus of vGAT-IRES-Cre transgenic mice for inhibitory neuron-specific expression^20^. The virus was highly expressed in the CA areas (CA3 – CA1), but not in the dentate gyrus (Fig. 1a). To examine the effects of inhibitory neuron-specific expression of KRAS^G12V^ on hippocampal-dependent spatial learning and memory, we performed the hidden-platform version of the Morris water maze (MWM) test. The vGAT-Cre::KRAS^G12V^ mice showed a significantly longer latency to find the hidden platform during the training sessions (Fig. 1b). In the probe test after 7 days of training, vGAT-Cre::KRAS^G12V^ mice spent significantly less time in the target quadrant in which the platform was located during the training sessions compared to the vGAT-Cre::EYFP mice (Fig. 1c). Furthermore, vGAT-Cre::KRAS^G12V^ mice swam significantly farther from the platform and cross the platform fewer times than vGAT-Cre::EYFP mice, suggesting that KRAS^G12V^ expression in vGAT^+^ inhibitory neurons in the adult hippocampus is sufficient to cause spatial learning and memory deficits (Supplementary Fig. S1a and b). Importantly, both groups showed comparable swimming speeds during the probe test in the hidden-platform version of the MWM test and performed well in the visible platform version of the MWM, suggesting that KRAS^G12V^ expression does not impair visuomotor functions (Supplementary Fig. S1c and d). However, open field analyses revealed that KRAS^G12V^ expression in vGAT^+^ inhibitory neurons increased total distance traveled, while anxiety-like behaviours assessed by the time spent in the center zone remained unchanged (Supplementary Fig. S1e and f).

Next, vGAT-Cre::KRAS^G12V^ mice were subjected to the object-place recognition test, another hippocampus-dependent task^21^. One day after training, vGAT-Cre::EYFP mice showed a preference for the relocated object, whereas vGAT-Cre::KRAS^G12V^ mice did not (Fig. 1d). Classical fear conditioning is another widely used Pavlovian learning and memory task in which animals associate non-aversive conditioned stimuli, such as context or an auditory tone, with aversive unconditioned stimuli, such as an electric foot shock^22^. The amygdala is critically involved in fear conditioning, particularly in auditory fear conditioning^22^. Contextual and auditory fear memory were assessed by measuring freezing behaviour when the trained mice were exposed to the same context or the same tone. vGAT-Cre::EYFP and vGAT-Cre::KRAS^G12V^ mice showed comparable levels of freezing in both contextual and auditory tests which were performed 1 and 2 days after training, respectively (Fig. 1e,f). These results show that the constitutive activation of KRAS in vGAT^+^ hippocampal neurons impairs spatial learning and memory in adult mice.

### Ectopic KRAS^G12V^ expression increases ERK activation in the inhibitory neurons

The G12V mutation dramatically decreases the conversion of GTP-bound KRAS to GDP-bound KRAS leading to hyperactivated RAS-ERK signalling^14^. To test whether KRAS^G12V^ expression increases the activation of RAS-ERK signalling in the adult hippocampal inhibitory neurons, we examined ERK phosphorylation (pERK1/2), a well-known marker for ERK activity, in the KRAS^G12V^ expressing neurons (Fig. 1g). We found that the percentage of pERK1/2-positive neurons was significantly higher in the HA-KRAS^G12V^ expressing inhibitory neurons compared to EYFP-expressing inhibitory neurons, suggesting that ectopic expression of KRAS^G12V^ could activate RAS-ERK signalling in inhibitory neurons (Fig. 1h). The total number of the virus-expressing cells was not different across groups (Supplementary Fig. S2).

### Inhibitory synaptic transmission is increased in the inhibitory neuron-specific KRAS^G12V^ expressing mice

**Figure 2:**
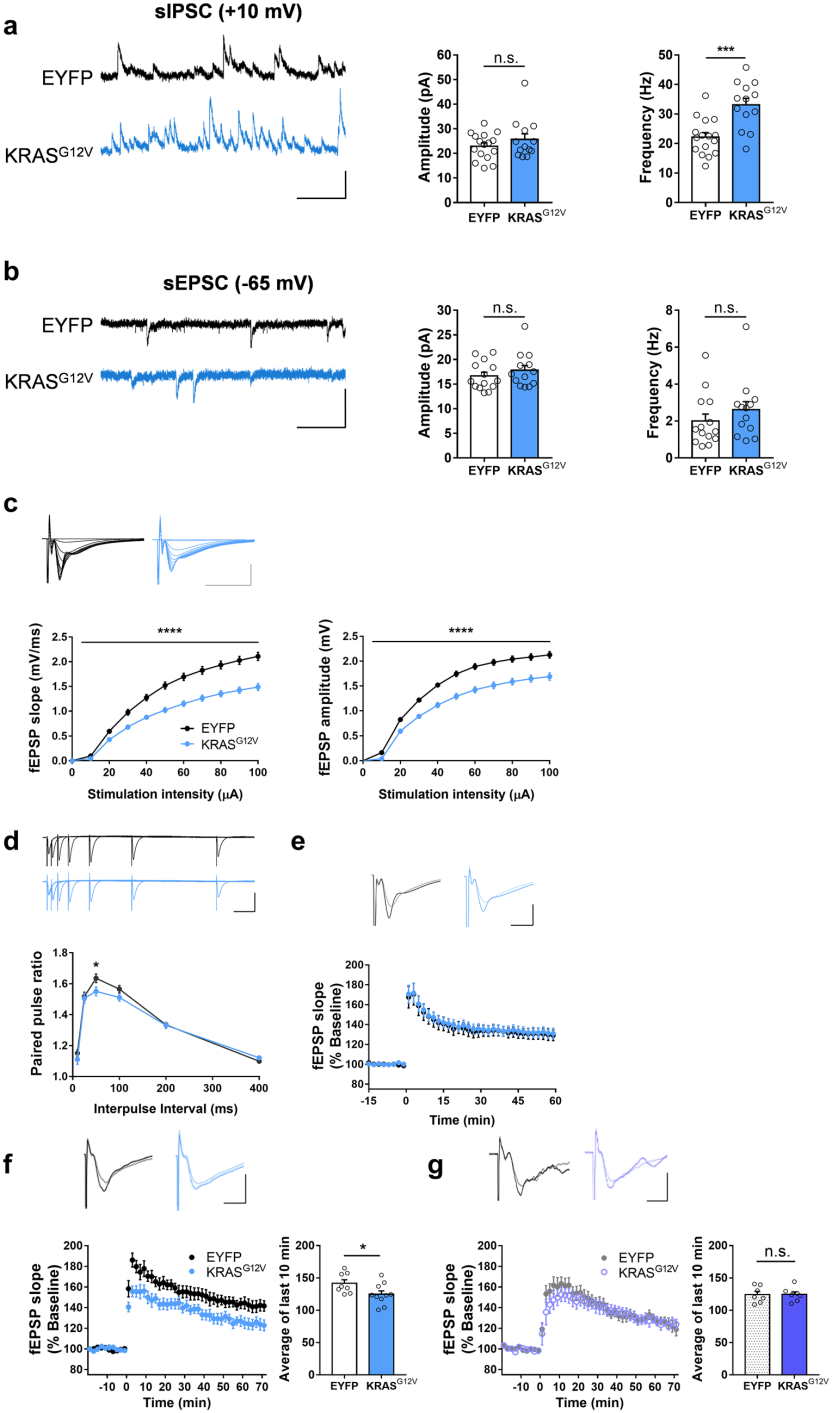
Ectopic KRAS^G12V^ expression in the inhibitory neurons increases inhibitory synaptic transmission and impairs LTP. (**a**) Ectopic KRAS^G12V^ expression in the inhibitory neurons increased the frequency of spontaneous inhibitory postsynaptic current (sIPSC). The amplitudes of the sIPSCs in the vGAT-Cre::KRAS^G12V^ mice were comparable to that from vGAT- Cre::EYFP mice (EYFP, n = 16 cells from 3 mice; KRAS^G12V^, n = 13 cells from 3 mice, unpaired t-test, *P* = 0.2883). The frequencies of the sIPSCs in the vGAT-Cre::KRAS^G12V^ mice were higher than that in the vGAT-Cre::EYFP mice (EYFP, n = 16 cells from 3 mice; KRAS^G12V^, n = 13 cells from 3 mice, unpaired t-test, ***P = 0.0003). Vertical bar, 50 pA; horizontal bar, 200 ms. (**b**) Ectopic expression of KRAS^G12V^ in the inhibitory neurons did not affect either the frequency or the amplitude of spontaneous excitatory postsynaptic current (sEPSC). The amplitudes of the sEPSCs were comparable between vGAT-Cre::KRAS^G12V^ and vGAT- Cre::EYFP; (EYFP, n = 14 cells from 3 mice; KRAS^G12V^, n = 13 cells from 3 mice, unpaired t-test, *P* = 0.3528). The frequency of the sEPSCs from the vGAT-Cre::KRAS^G12V^ were similar with that from vGAT-Cre::EYFP; KRAS^G12V^ (EYFP, n = 14 cells from 3 mice; KRAS^G12V^, n = 13 cells from 3 mice, unpaired t-test, *P* = 0.3044). Vertical bar, 50 pA; horizontal bar, 200 ms. (**c**) Ectopic KRAS^G12V^ expression in the inhibitory neurons significantly decreased the input–output relationship of the field excitatory postsynaptic potential (fEPSP) at CA3-CA1 synapse. Outputs were measured either by initial fEPSP slope (left) or peak amplitude (right). vGAT-Cre::EYFP, n = 20 slices from 10 mice; vGAT-Cre::KRAS^G12V^, n = 23 slices from 10 mice; Two-way repeated measures ANOVA, effect of virus, ****P < 0.0001 for both slope and amplitude. Vertical bar, 1 mV; horizontal bar, 10 ms. (**d**) Paired pulse facilitation ratio was significantly decreased at 50 ms interpulse interval in vGAT-Cre::KRAS^G12V^mice. vGAT-Cre::EYFP, n = 20 slices from 10 mice; vGAT-Cre::KRAS^G12V^, n = 23 slices from 10 mice, unpaired t-test, *P = 0.0403. Vertical bar, 1 mV; horizontal bar, 50 ms. (**e**) Ectopic KRAS^G12V^ expression in inhibitory neurons did not affect long-term potentiation (LTP) induced by weak stimulation protocol (4 bursts of 4 100 Hz pulses, 5 Hz inter-burst interval). vGAT-Cre::EYFP, n = 10 slices from 5 mice; vGAT-Cre::KRAS^G12V^, n = 12 slices from 5 mice. Traces represent the average of fEPSPs at the baseline (-15 min – 0 min) and after LTP induction (50 – 60 min). Vertical bar, 1 mV; horizontal bar, 5 ms. (**f**) Ectopic KRAS^G12V^ expression in inhibitory neurons significantly impaired LTP induced by the stronger stimulation protocol (10 bursts of 4 100 Hz pulses, repeated 4 times with 10 s interval). vGAT-Cre::EYFP, n = 8 slices from 5 mice; vGAT-Cre::KRAS^G12V^, n = 10 slices from 5 mice. The averages fEPSP slope of 61 to 70 min after LTP induction were compared. Unpaired t test, *P = 0.0422. Traces represent the average of fEPSPs at the baseline (-20 min – 0 min) and after LTP induction (61 – 70 min). Vertical bar, 1 mV; horizontal bar, 5 ms. (**g**) Picrotoxin (10 μM) treatment rescued the strong TBS-induced LTP deficit at CA3-CA1 synapse in vGAT-Cre::KRAS^G12V^ mice. vGAT-Cre::EYFP, n = 7 slices from 3 mice; vGAT-Cre::KRAS^G12V^, n = 7 slices from 3 mice. The averages fEPSP slope of 61 to 70 min after LTP induction were compared. Unpaired t test, *P* = 0.9887. Traces represent the average of fEPSPs at the baseline (-20 min – 0 min) and after LTP induction (61 – 70 min). Vertical bar, 1 mV; horizontal bar, 5 ms. Data are expressed as the mean ± SEM.

To assess whether the overexpression of KRAS^G12V^ in inhibitory neurons affects their electrophysiological properties, we measured spontaneous inhibitory postsynaptic currents (sIPSC) in the hippocampal pyramidal neurons in vGAT-Cre::KRAS^G12V^ mice using whole-cell patch-clamp recordings. We found that the sIPSC frequency was significantly higher in vGAT-Cre::KRAS^G12V^ mice than in vGAT-Cre::EYFP mice (Fig. 2a). Ectopic expression of KRAS^G12V^ in vGAT^+^ inhibitory neurons did not affect the sIPSC amplitude (Fig. 2a). The frequency and amplitude of the spontaneous excitatory postsynaptic currents (sEPSC) in the KRAS^G12V^ group were comparable to those of the EYFP group (Fig. 2b). These data show that the activation of KRAS-ERK pathway enhances GABAergic synaptic transmission. Next, we examined whether the changes in GABAergic synaptic transmission affects synaptic plasticity in the hippocampus. We recorded field excitatory postsynaptic potentials (fEPSPs) in the hippocampal CA3-CA1 Schaffer-collateral pathway. We found that the inhibitory neuron-specific expression of KRAS^G12V^ significantly decreased the input–output relationship of fEPSPs (Fig. 2c). Both the initial slope and amplitude of fEPSP were decreased in vGAT-Cre::KRAS^G12V^ (Fig. 2c). The paired-pulse facilitation ratio was significantly decreased at 50 ms of inter-stimuli interval in vGAT-Cre::KRAS^G12V^ comparison to vGAT-Cre::EYFP (Fig. 2d). Long-term potentiation (LTP) is considered as a cellular mechanism for memory and LTP impairments have been frequently observed in mutant mice with learning and memory deficits including RASopathy models^16,23^. However, a weak theta-burst stimulation (TBS; 4 bursts of 4 100 Hz pulses, 5 Hz inter-burst interval)-induced LTP was normal in the hippocampus of vGAT-Cre::KRAS^G12V^ mice (Fig. 2e). When LTP was induced by the stronger stimulation protocol (10 bursts of 4 100 Hz pulses, repeated 4 times with 10 s interval), we found that LTP is significantly reduced in vGAT-Cre::KRAS^G12V^ in comparison with that in vGAT-Cre::EYFP (Fig. 2f). To assess whether the LTP deficit is caused by increased inhibitory function, we induced LTP with the strong protocol in the presence of a GABA_A_ receptor antagonist picrotoxin (10 µM). Indeed, picrotoxin treatment rescued LTP deficits in vGAT-Cre::KRAS^G12V^ mice (Fig. 2g). Taken together, our results suggest that inhibitory neuron-specific expression of KRAS^G12V^ induces deficits in synaptic plasticity via increased basal inhibitory synaptic transmission.

### Ectopic expression of KRAS^G12V^ in the hippocampal excitatory neurons impairs spatial learning and memory in mice

**Figure 3:**
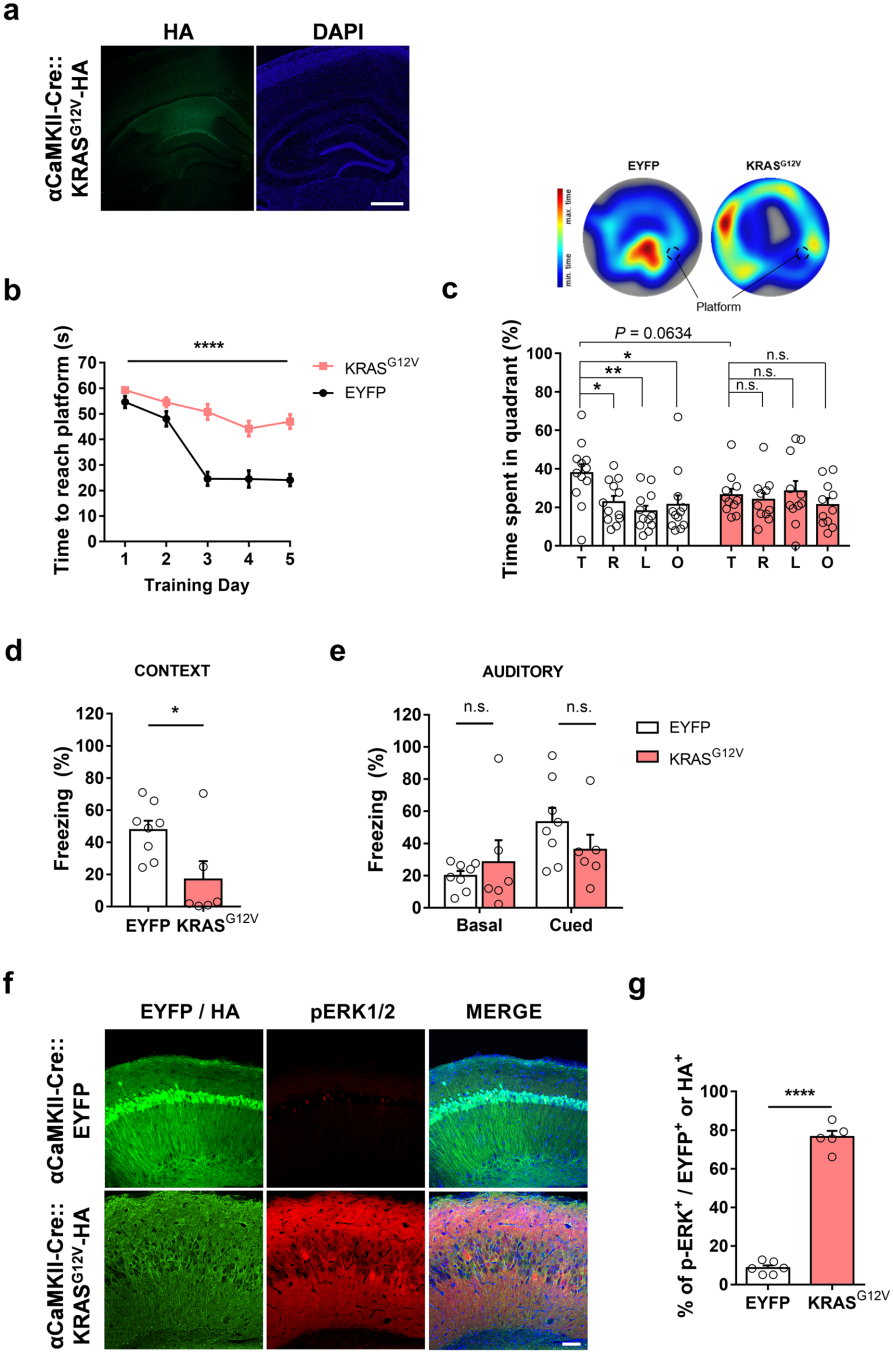
Ectopic expression of KRAS^G12V^ in excitatory neurons impairs spatial memory and induces ERK activation. (**a**) HA staining of KRAS^G12V^-expressing hippocampal slices from αCaMKII-Cre mice. 4′,6-diamidino-2-phenylindole (DAPI) staining was used to identify nuclei. Scale bars, 200 μm. (**b**) Learning is significantly slower in KRAS^G12V^ expressing mice compared to EYFP controls. αCaMKII-Cre::EYFP, n = 12; αCaMKII-Cre::KRAS^G12V^, n = 11; Two-way repeated measures ANOVA, F_1, 21_ = 69.63, ****P < 0.001 (**c**) Lower panel, time spent in each quadrant during the probe test. αCaMKII-Cre::EYFP mice selectively searched for the platform in the target quadrant, while KRAS^G12V^ did not. αCaMKII-Cre::EYFP, n = 12, One-Way ANOVA, followed by a Dunnett’s post hoc test, *P = 0.0292, **P = 0.0030, *P = 0.0156; αCaMKII-Cre::KRAS^G12V^, n = 11, One-way ANOVA, followed by Dunnett’s post hoc test, *P* = 0.9510, *P* = 0.9743, *P* = 0.7006. αCaMKII-Cre::KRAS^G12V^ mice tend to spend less time in the target quadrant compared to EYFP mice (unpaired t-test, *P* = 0.0634). T, target quadrant, R, right to target, L, left to target, O, opposite to target. Upper panel, representative heat map summary of MWM probe test. Platform position during the training trials is indicated by the dotted circle. (**d**) In contextual fear memory test, αCaMKII-Cre::KRAS^G12V^ mice showed significantly reduced freezing behaviour compared to αCaMKII-Cre::EYFP mice. αCaMKII-Cre::EYFP, n = 8; αCaMKII-Cre::KRAS^G12V^, n = 6; unpaired t-test, *P = 0.0247. (**e**) The freezing level of αCaMKII-Cre::KRAS^G12V^ in response to the conditioned tone (cue) was not statistically different from that of αCaMKII-Cre::EYFP mice. αCaMKII-Cre::EYFP, n = 8; αCaMKII-Cre::KRAS^G12V^, n = 6; unpaired t-test, *P* = 0.5026 for basal freezing level before tone played, *P* = 0.2178 for cued freezing. (**f**) Representative images of immunohistochemistry from slices expressing EYFP or KRAS^G12V^ in excitatory neurons. Slices were immunostained for HA (green), p-ERK1/2 (red), and DAPI (blue). Scale bar = 80 μm. (**g**) The percentage of pERK-positive neurons was significantly higher in KRAS^G12V^expressing hippocampal slices. αCaMKII-Cre::EYFP, n = 6 slices from 3 hippocampi, αCaMKII-Cre::KRAS^G12V^, n = 5 slices from 3 hippocampi, unpaired t-test, ****P < 0.0001. Data are expressed as the mean ± SEM.

In adult mice, *Kras* is expressed not only in inhibitory neurons but also in excitatory neurons^19^. To investigate the effects of ectopic KRAS^G12V^ expression in the excitatory neurons on learning and memory, we injected AAV-KRAS^G12V^–HA into the dorsal hippocampus of αCaMKII-Cre mice (Fig. 3a), thereby allowing the selective expression of KRAS^G12V^ in the excitatory neurons^24^. We then subjected the mice to the MWM test. αCaMKII-Cre::KRAS^G12V^ mice showed a significantly increased escape latency during the training sessions of the MWM test, suggesting that KRAS^G12V^ expression in the excitatory neurons severely impaired spatial learning (Fig. 3b). In probe trials, αCaMKII-Cre::KRAS^G12V^ mice failed to identify the target quadrant, whereas the control αCaMKII-Cre::EYFP mice spent a significantly longer time in the target quadrant, suggesting that KRAS^G12V^ expression in the adult hippocampal excitatory neurons also impairs spatial memory (Fig. 3c). Moreover, αCaMKII-Cre::KRAS^G12V^ mice swam farther from the platform location (Supplementary Fig. S3a) and crossed the platform fewer times than the EYFP-expressing control mice (Supplementary Fig. S3b). Of note, αCaMKII-Cre::KRAS^G12V^ mice swam significantly slower than αCaMKII-Cre::EYFP mice during the probe trials and showed an increased latency to find the platform in the initial trials of the visible platform version of the MWM. However, they performed well in the later trials, suggesting that expressing KRAS^G12V^ in hippocampal inhibitory neurons may also affect locomotive behaviours (Supplementary Fig. S3c and d). However, even when we analysed the path length to the platform during the training sessions in the hidden platform version of the MWM, αCaMKII-Cre::KRAS^G12V^ mice took longer path to the platform than αCaMKII-Cre::EYFP mice, demonstrating that the learning deficits in the αCaMKII-Cre::KRAS^G12V^ mice cannot be attributed to the altered locomotive behaviours (Supplementary Fig. S3e). Furthermore, αCaMKII-Cre::KRAS^G12V^ mice showed significantly lower freezing time compared to αCaMKII-Cre::EYFP mice in contextual test, but not in auditory test in the classical fear conditioning (Fig. 3d,e), showing that expressing KRAS^G12V^ in excitatory neurons impairs hippocampus-dependent memory without affecting amygdala-dependent memory. Interestingly, αCaMKII-Cre::KRAS^G12V^ mice showed hyperactivity and avoided the center zone in the open field test (Supplementary Fig. S3f and g), suggesting that the behavioural deficits in αCaMKII-Cre::KRAS^G12V^ mice are not limited to learning and memory.

### Ectopic expression of KRAS^G12V^ in the excitatory neurons increases ERK activity and induces neuronal cell death

**Figure 4:**
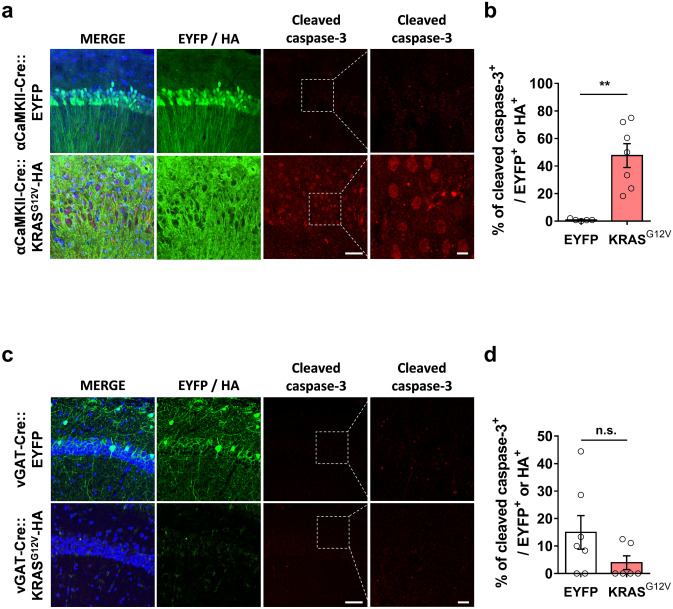
KRAS^G12V^ induced neuronal cell death only in excitatory neurons. (**a**) αCaMKII-Cre mice were injected with either AAV expressing EYFP or HA-KRAS^G12V^. Slices were immunostained for cleaved caspase-3 (red), HA (green), and DAPI (blue), Scale bar = 50 μm; high magnification, Scale bar = 20 μm. (**b**) The percentage of cleaved caspase-3 positive neurons was significantly higher in the KRAS^G12V^expressing hippocampal slices. αCaMKII-Cre::EYFP, n = 5 slices from 3 hippocampi, αCaMKII-Cre::KRAS^G12V^, n = 7 slices from 3 hippocampi, unpaired t-test, **P = 0.0012. (**c**) vGAT-Cre mice were injected with either AAV expressing EYFP or HA- KRAS^G12V^. Slices were immunostained for cleaved caspase-3 (red), HA (green), and DAPI (blue), Scale bar = 50 μm, high magnification; Scale bar = 20 μm. (**d**) The percentage of cleaved caspase-3 positive neurons was comparable between vGAT-CRE::EYFP and vGAT-Cre::KRAS^G12V^mice; vGAT-CRE::EYFP, n = 6 slices from 3 hippocampi, vGAT-Cre::KRAS^G12V^, n = 6 slices from 4 hippocampi, unpaired t-test, n.s., not significant, *P* = 0.2488. Data are expressed as the mean ± SEM.

We observed that ectopic expression of HA-tagged KRAS^G12V^ in the hippocampal CA1 region of αCaMKII-Cre mice caused hyperactivation of RAS-ERK signalling as accessed by pERK immunostaining (Fig. 3f,g). Interestingly, we also observed that the hippocampal CA1 region showed morphological alterations such as the decrease in cell density and the expansion of the pyramidal layer (Fig. 3f). To visualize the morphological abnormalities, we performed Nissl staining and immunohistochemistry on hippocampal slices from EYFP or KRAS^G12V^ expressing mice (Supplementary Fig. S4). The pyramidal cell layer was distended, but the dentate gyrus, where the mutant protein was not expressed, appeared normal (Supplementary Fig. S4). This morphological alteration might have been due to cell death. To test whether ectopic KRAS^G12V^ expression induced neuronal cell death, we examined the expression of cleaved caspase-3, which is an apoptosis-dependent cell death marker^25,26^. The number of cleaved caspase-3 positive neurons was significantly increased in the αCaMKII-Cre::KRAS^G12V^ mice compared to αCaMKII-Cre::EYFP mice (Fig. 4a,b). Moreover, we observed that ectopic KRAS^G12V^ expression did not induce apoptosis in inhibitory neurons (Fig. 4c,d).

## Discussion

Herein, we report that ectopic expression of a gain-of-function KRAS mutation in either excitatory or in inhibitory neurons in the adult hippocampus increases ERK activation and impairs spatial learning and memory in mice. In this study, we used a strong G12V KRAS mutation to mimic severe cases of RASopathies caused by strong *KRAS* mutations such as G12S to investigate the cellular mechanisms of cognitive deficits in these RASopathies.

### Cell type specific roles of RAS signalling in learning and memory

It has been shown that the RAS proteins are critically involved in learning and memory and synaptic plasticity^16,27^. Hyperactivation as well as hypoactivation of RAS-ERK signalling impairs learning and memory and synaptic plasticity^16,27–29^. More interestingly, mutations in the essential genes of RAS-ERK signalling cascade have cell type-specific effects on the downstream signalling activation as well as synaptic functions. *Nf1* haploinsufficiency affects only GABAergic synaptic transmission in the cortex and the hippocampus^30,31^. Consistently, inhibitory neuron-specific deletion of *Nf1* recapitulated learning deficits of *Nf1*^+/-^ mice^30^. Cell type-specificity has also been reported for excitatory neurons. SHP2^D61G/+^ mice showed increased level of ERK activation only in excitatory neurons and enhanced glutamatergic synaptic transmission but no changes in inhibitory synaptic functions^32^. Ectopic expression of SHP2^D61G^ selectively in the excitatory but not in inhibitory neurons increased ERK activation and impaired spatial learning and LTP^19^. One explanation for these cell type-specific effects of RAS signalling is that the expression profiles of RAS-ERK signalling genes are significantly different between hippocampal excitatory and inhibitory neurons^19^. The *NF1* transcript is significantly enriched in inhibitory neurons compared to excitatory neurons in both mice as well as in humans, which may account for its critical roles in regulating inhibitory synaptic transmission^19,33^. Similarly, *Kras* mRNA expression was shown to be significantly higher in inhibitory neurons than excitatory neurons in the adult mouse hippocampus, suggesting that *Kras* mutations may have a more severe impact on inhibitory neurons^19^. Consistent with this hypothesis, a heterozygous knock-in mouse expressing KRAS^G12V^ under the control of a synapsin promoter (Syn-cre; KRAS^G12V^ mice) showed increased inhibitory synaptic functions and deficits in LTP and spatial memory^18^. Moreover, inhibitory neuron-specific expression of KRAS^G12V^ also increased the GABAergic synaptic area and impaired the memory functions, demonstrating the critical role of KRAS^G12V^ in inhibitory neurons^18^. Consistently, our results also show that KRAS is involved in regulating GABA release in inhibitory neurons. Interestingly, we found that KRAS^G12V^ induces cell death only in excitatory neurons, and not in inhibitory neurons, thereby demonstrating that KRAS also plays cell type specific roles in the nervous system.

### Role of KRAS in inhibitory neurons

Papale et al. showed that ERK hyperactivation is only observed in the early phases of postnatal development (P10 and P21) in Syn-cre;KRAS^G12V^ mice^18^. They also reported that reducing GABAergic inhibition but not suppressing ERK hyperactivation rescued the LTP and memory deficits in the adult mutants, suggesting that increased RAS-ERK signalling mainly accounts for the developmental defects such as increased inhibitory synaptogenesis^18^. However, the effects of cell-type specific activation of ERK signalling in the adult hippocampus have not been fully examined. Moreover, it remained unclear whether KRAS^G12V^ in mature neurons contributes to the behavioural and physiological deficits in Syn-cre;KRAS^G12V^ mice in which KRAS^G12V^ is expressed in all types of neurons. In this study, we found that KRAS^G12V^ increases ERK activation in the adult hippocampal inhibitory neurons, which is sufficient to cause deficits in synaptic plasticity and spatial memory. Our results demonstrate that KRAS^G12V^-induced increase in ERK activity in inhibitory neurons is at least partially responsible for the behavioural deficits in the adult mice, which can be exploited for developing alternative treatment strategies for targeting RASopathies.

The RAS-ERK signalling cascade regulates GABA release by phosphorylating synapsin at the inhibitory synapse^30^. Consistently, we also observed that expressing KRAS^G12V^ in inhibitory neurons increases ERK activation and sIPSC frequency. However, a detailed molecular mechanism how KRAS^G12V^ increases the spontaneous GABA release remains to be investigated. We also found that the input–output curve of fEPSP at the CA3-CA1 synapse is significantly suppressed by expressing KRAS^G12V^ in inhibitory neurons, which might be caused by the constitutively increased inhibitory tone. It has been shown that dendritic inhibition is effective at shunting postsynaptic depolarization^34^.

It has been shown that increased GABAergic synaptic transmission can impair LTP^18,30,35^. Interestingly, LTP was not impaired in vGAT-Cre::KRAS^G12V^ mice when we used a weak stimulation protocol for LTP induction which was also used in Syn-cre;KRAS^G12V^ mice exhibiting LTP deficits^18^. Differences in mouse models such as the generation methods (knock-in versus viral overexpression, synapsin promotor versus vGAT promoter) may account for this discrepancy. However, when we used the stronger stimulation protocol (4 episodes of 10 TBS) which should activate more inhibitory synapses^35^, we were able to reveal significant LTP deficits in vGAT-Cre::KRAS^G12V^ mice. Picrotoxin treatment rescued the LTP deficit, suggesting that the increased inhibition is responsible for the LTP deficits. Taken together, our results suggest that KRAS in adult vGAT^+^ neurons is critically involved in regulating GABA release, and that its dysregulation results in deficits in synaptic plasticity and memory.

### Role of KRAS in excitatory neurons

Next, we assessed whether excitatory neurons also contribute to the phenotypes in *KRAS* mutations-associated neurodevelopmental disorders. We observed that, similar to inhibitory neurons, the ectopic expression of KRAS^G12V^ specifically in excitatory neurons also resulted in severe spatial learning and memory deficits in adult mice. Interestingly, KRAS^G12V^ expression induced neuronal cell death selectively in excitatory neurons, but not in inhibitory neurons. Thus, neuronal apoptosis in KRAS^G12V^ expressing mice prevented us from analysing the electrophysiological properties in these mice. Moreover, KRAS^G12V^ expression induced histological changes such as an expanded pyramidal cell layer. Not surprisingly, αCaMKII-Cre::KRAS^G12V^ mice showed complex behavioural phenotypes such as increased locomotive activity and anxiety-like behaviours in the open field test, which may also contribute to the learning and memory phenotypes. Consistent with our findings, it has been shown that nestin-derived KRAS^G12V^ expression causes apoptosis in neural progenitor cells, leading to severe brain oedema in zebrafish^36^. Although RAS-ERK signalling is well known for its anti-apoptotic or pro-survival functions, it also has pro-apoptotic functions^37^. For example, in several conditions such as focal cerebral ischemia, DNA damaging stimuli and oxidative toxicity promote cell death through ERK signalling^37^. Moreover, it was shown that ERK activation is required for caspase-3 activation^38^. Activation of ERK signalling may elicit either apoptosis or cell survival depending on conditions, the mechanisms of which are not fully known. In addition, the mechanisms underlying KRAS-induced apoptosis remain unknown. In addition, it remains to be investigated how KRAS^G12V^ induces apoptosis only in excitatory neurons, but not in inhibitory neurons. As previously shown, since RAS signalling networks are different between excitatory and inhibitory neurons, we speculate that KRAS-ERK signalling pathway is not coupled to pro-apoptotic signalling in inhibitory neurons.

Previous studies have shown that mutations in genes involved in the RAS signalling pathway can alter the composition of cell types in both the developing and adult brain^16,39,40^. Changes in the ratio of excitatory and inhibitory neurons can subsequently distort the excitation-inhibition balance, which is critical for normal cognitive functions, including learning and memory^41,42^. Excitatory neuron-specific cell death in adults, which contribute to behavioural deficits, might have been overlooked and may also contribute to the increased inhibition to excitation ratio which has been reported in other RASopathy models^16,18,30,32^. However, we cannot completely exclude the possibility that the disrupted cellular organization in CA1, including the enlarged intercellular space in CA regions, might also have affected the synaptic connectivity in the hippocampus, thereby contributing to behavioural deficits.

Taken together, our study’s results showed that a gain-of-function *KRAS* mutation affects the function and survival of inhibitory and excitatory neurons, respectively. Further, this may have synergistic effects on cognitive deficits in RASopathies (Supplementary Fig. S5). Finally, our findings may contribute to better understanding the physiological roles of KRAS in adult neurons.

## Methods

### Mice

αCaMKII-Cre (JAX 005,359) and vGAT-IRES-Cre (JAX 016,962) mice were maintained by breeding with wild-type C57Bl/6 J mice at the SNU Specific Pathogen Free centre. Animals were group-housed (four mice per cage) on a 12-h light/dark cycle in the vivarium at SNU. Both female and male mice (2–7 months old) were used for all experiments. All studies were approved by the Seoul National University Institutional Animal Care and Use Committee. All experiments were performed in accordance with the institutional guidelines and regulations.

### AAV packaging

AAV packaging was performed as described previously^19^. Briefly, EYFP or hemagglutinin (HA) tagged-KRAS^G12V^ were inserted into the pAAV-EF1a-DIO-WPRE plasmid. For AAV packaging, HEK 293 T cells (7 × 10^12^) were cultured on dishes (Thermo Fisher 157,150) with 15 mL DMEM (Thermo Fisher Scientific SH30243.01) containing 10% fetal bovine serum (Thermo Fisher Scientific SH30919.03) and penicillin/streptomycin (GIBCO 15,140–122) at 37 °C and 5% CO_2_. p5E18-RxC1 (13 μg), pAd-ΔF6 plasmid (26 μg), and pAAV plasmid (EYFP or KRAS^G12V^; 13 μg) were transfected into HEK 293 T cells using polyethylenimine (PEI, Polysciences, Inc.) transfection method. The transfection medium was replaced with 20 mL fresh medium. After 72 h, the culture medium was harvested for AAV purification. The supernatants were layered in an ultracentrifuge tube (Beckman 324,214) in the following order: culture medium from each dish, 6 mL of 15% iodixanol (Opti-Prep; Axis-shield 1045) solution [1 M NaCl, 1 mM MgCl_2_, 2.5 mM KCl, and 25% Opti-prep in phosphate-buffered saline (PBS)], 5 mL of 25% solution (2.5 mM KCl, 0.2% phenol red, 1 mM MgCl_2_, and 42% Opti-prep in PBS), 5 mL of 40% solution (2.5 mM KCl, 1 mM MgCl_2_ and 67% Opti-prep in PBS) and 4 mL of 60% solution (2.5 mM KCl, 1 mM MgCl_2_, and 0.2% phenol red in Opti-prep). The iron seared tubes were centrifuged at 69,000 rpm for 1 h using the Beckman UltimaTL-100 K ultracentrifuge and 70Ti rotor. The 40% solution was harvested from the centrifuged column using a syringe. The harvested solution was diluted with PBS, filtered with the Amicon ultra-15 filter tube (Millipore UFC910024). The filter was washed 3 times with 15 mL PBS. The viral particles in the solution were quantified via qPCR.

### Stereotaxic viral injection

Stereotaxic surgery was performed as previously described^19^. Briefly, AAV (1 µl of 4.8 × 10^12^ vg/mL) was injected into the dorsal hippocampal CA1 region of the 8 weeks old male mice at the following stereotactic coordinates from the bregma: anterior/posterior (AP): -1.8 mm, medial/lateral (ML): ± 1.0 mm, dorsal/ventral (DV): -1.7 mm / AP: -2.5 mm, ML: ± 2.0 mm, DV: -1.8 mm. All mice were allowed to recover for a period of minimum 3–4 weeks. The investigators were blinded to the types of viral vectors injected in each mice.

### Morris water maze (MWM) test

The MWM test was performed as previously described^19^. Briefly, mice were handled for 2 min at the same time every day for seven consecutive days before the test. The maze consisted of a non-transparent cylindrical tank (diameter, 120 cm) in a room with visual cues, which are the animal's navigational references for locating the platform. The tank was filled with water (21–22 °C) and painted white. The tank was divided into four invisible quadrants (target quadrant, T; opposite quadrant, O; right quadrant, R; left quadrant, L.) and a platform, which was submerged 1 cm under the surface of the water, was placed at the centre of the target quadrant. Before the first trial on training day 1, each mouse was placed onto the platform for 30 s. On training days, mice were placed at the start position, chosen randomly for each trial. Mice were trained with six trials per day. Probe tests were performed under the same conditions as the training trials, except that the platform was absent in the test session. In the probe tests, the mice were tracked for 1 min using a tracking program (EthoVision 11.5; Nodulus). For vGAT-Cre::KRAS^G12V^ and vGAT-Cre::EYFP mice, the probe test was performed on day 7 after the training. For CaMKII-Cre::KRAS^G12V^ and CaMKII-Cre::EYFP mice, the probe test was performed 5 days after the training. The visible platform-version of MWM was performed after the hidden-platform test. The platform was tagged with a visible flag. The visible version of water maze was performed after the hidden-platform test. Latency to reach the flagged platform were assessed.

### Object-place recognition (OPR) test

OPR test was performed as described previously^19^. Mice were handled for 5 min for four consecutive days and habituated in a cube-shaped acrylic box (32 cm by 32 cm by 32 cm) for 15 min for another 2 days before performing the training and test. In the training session, mice were placed in the box containing two identical 100 ml glass bottles and were allowed to explore the objects for 10 min. In the test session, 24 h after training, mice were placed in the same box containing one object that stayed in the same location and the other object relocated to a new position. All locations for the objects were counterbalanced among groups, and objects were cleaned between trials. Sessions were recorded and later analyzed manually. Experimenters were blinded to the type of injected viral vectors.

### Classical fear conditioning

Classical fear conditioning test was performed as previously described^43^. Mice were placed in the conditioning chamber for 2 min before the delivery of three times of paired stimuli containing a tone (2800 Hz, 85 dB, 30 s) and a co-terminating electric foot shock (2 s, 0.4 mA). Contextual fear memory was assessed 24 h after training, in which the percentage of time mice spent freezing was measured in the same chamber where they were previously shocked. In cued fear memory test performed 1 h or 1 day after the contextual memory test, mice were placed in a novel context for 2 min followed by the conditioned tone for 3 min. The percentage of freezing time was automatically scored by Freeze Frame software (ActiMetrics, IL., USA).

### Immunohistochemistry

Immunohistochemistry was performed as described previously^19^. Briefly, the mice hippocampi were fixed in 4% paraformaldehyde (Sigma-Aldrich P6148) containing 20 mM Na3VO4 and 100 mM NaF for 24 h and incubated in 30% sucrose-PBS for 2 days. The fixed brain samples were frozen and stored at -80 °C until sectioning. Brain slices (20 μm thick) were sectioned using a cryostat and stored in a cold 50% glycerol PBS or CPS solution (25% glycerol, 25% ethylene glycol, and 50% 0.1 M PO4 buffer) at -20 °C. Brain sections were washed with PBS (5 min per wash) and transferred into the blocking solution [0.2% Triton-X 100 (Sigma-Aldrich T8787) and 4% goat serum (Rockland D104-00–0050) in PBS] for 1 h at room temperature. Sections were then incubated with primary antibody [anti-HA, 1:50 (Roche, 11,867,423,001); p-ERK1/2, 1:400 (Cell signalling, 9101S); or cleaved caspase-3, 1:400 (Cell signalling, 9661S)] in blocking solution for 48 h at 4 °C followed by incubation in secondary antibodies for 3 h at RT. Images were acquired on the LSM-700 confocal microscope (Carl Zeiss, Germany) with Zen software and analysed using ImageJ.

## Electrophysiology

### Field recording

Field EPSP recordings were performed as previously described^32^. Briefly, mouse brains were sliced into sagittal Sects. (400 μm thickness) using a vibratome (Campden Instruments, Oxford, UK) in ice-chilled slicing solution (2.5 mM KCl, 30 mM NaHCO_3_, 93 mM NMDG, 20 mM HEPES, 2 mM thiourea, 1.25 mM NaH_2_PO_4_, 10 mM MgSO_4_, 25 mM d-glucose, 5 mM sodium ascorbate, 3 mM sodium pyruvate, and 0.5 mM CaCl_2_, oxygenated with 5% CO_2_ and 95% O_2_). Slices were allowed to recover in the slicing solution at 32 °C for 15 min. Slices were incubated in ACSF (3.5 mM KCl, 1.25 mM NaH_2_PO_4_, 1.3 mM MgSO_4_, 120 mM NaCl, 2.5 mM CaCl_2_, 10 mM glucose, 20 mM NaHCO_3_, oxygenated at room temperature for at least 30 min before recording). Slices were translocated into a recording chamber and fEPSPs were recorded from the Schaffer collaterals in the CA3–CA1 pathway. A stimulation intensity of 30–40% of the maximum response was chosen for these studies. All the electrophysiological experiments were performed as previously described^32^. The input–output relationship was determined by measuring the fEPSP slope at stimulation intensities (0—100 μA). The paired-pulse facilitation ratio was analysed over increasing time intervals (10, 25, 50, 100, 200, and 400 ms). LTP was induced by either a weak theta-burst stimulation (TBS) protocol (4 bursts of stimuli delivered at 5 Hz; each burst contains four pulses at 100 Hz) or strong TBS protocol (4 episodes of TBS with 10 s interval; each episode consists of 10 TBS). Data were analysed using WinLTP software (WinLTP Ltd., Bristol, UK).

### Whole cell patch clamp recording

The whole-cell patch was performed as described previously^44^. Briefly, transverse hippocampal slices (300 μm) were prepared using a vibratome (Leica, VT1200S) in an ice-chilled slicing solution (2.5 mM KCl, 30 mM NaHCO_3_, 93 mM NMDG, 20 mM HEPES, 2 mM thiourea, 1.25 mM NaH_2_PO_4_, 10 mM MgSO_4_, 25 mM d-glucose, 5 mM sodium ascorbate, 3 mM sodium pyruvate, and 0.5 mM CaCl_2_, saturated with 5% CO_2_ and 95% O_2_). Slices were allowed to recover in the slicing solution at 32 °C for 30 min. The slices were transferred into an incubation chamber filled with ACSF solution saturated with 5% CO_2_ and 95% O_2_ and maintained for at least 1 h before recordings were made. The whole-cell patch-clamp recording was performed at 30 °C during continuous perfusion at 4 ml/min with ACSF. All recordings were performed using EPC 10 (HEKA Elektronik) with a sampling frequency of 20 kHz and the signals were filtered at 10 kHz. Patch pipettes (3–4 MΩ) were borosilicate glass filled with the internal solution (135 mM CsMS; 10 mM CsCl; 10 mM HEPES; 0.2 mM EGTA; 4 mM Mg-ATP; 0.4 mM Na2-GTP; 0.5 mM spermine; ~ 295 mOsm; pH 7.3 adjusted with CsOH). Experiments were only accepted for analysis if series resistance values were < 25 mega-ohms (± 20%) throughout the recordings. Cells were clamped at a holding potential of − 70 mV to measure the spontaneous excitatory postsynaptic current (sEPSC). Cells were clamped at a holding potential of + 10 mV to measure the spontaneous inhibitory postsynaptic current (sIPSC).

### Statistics

Statistical analyses were performed by using the GraphPad Prism 7.0 software. Groups were compared using an unpaired two-tailed t-test. Analysis of variance (ANOVA) was used for multiple group comparisons, followed by appropriate post-tests as indicated.

## Supplementary information

Supplementary Figure 1.

Supplementary Figure 2.

Supplementary Figure 3.

Supplementary Figure 4.

Supplementary Figure 5.

Supplementary Legend 6.
