# Supplementary figures and images for: Neuron type-specific expression of a mutant KRAS impairs hippocampal-dependent learning and memory
Source: Sci Rep. 2020 Oct 20;10:17730. doi: 10.1038/s41598-020-74610-y (PMC7575532; doi:10.1038/s41598-020-74610-y)

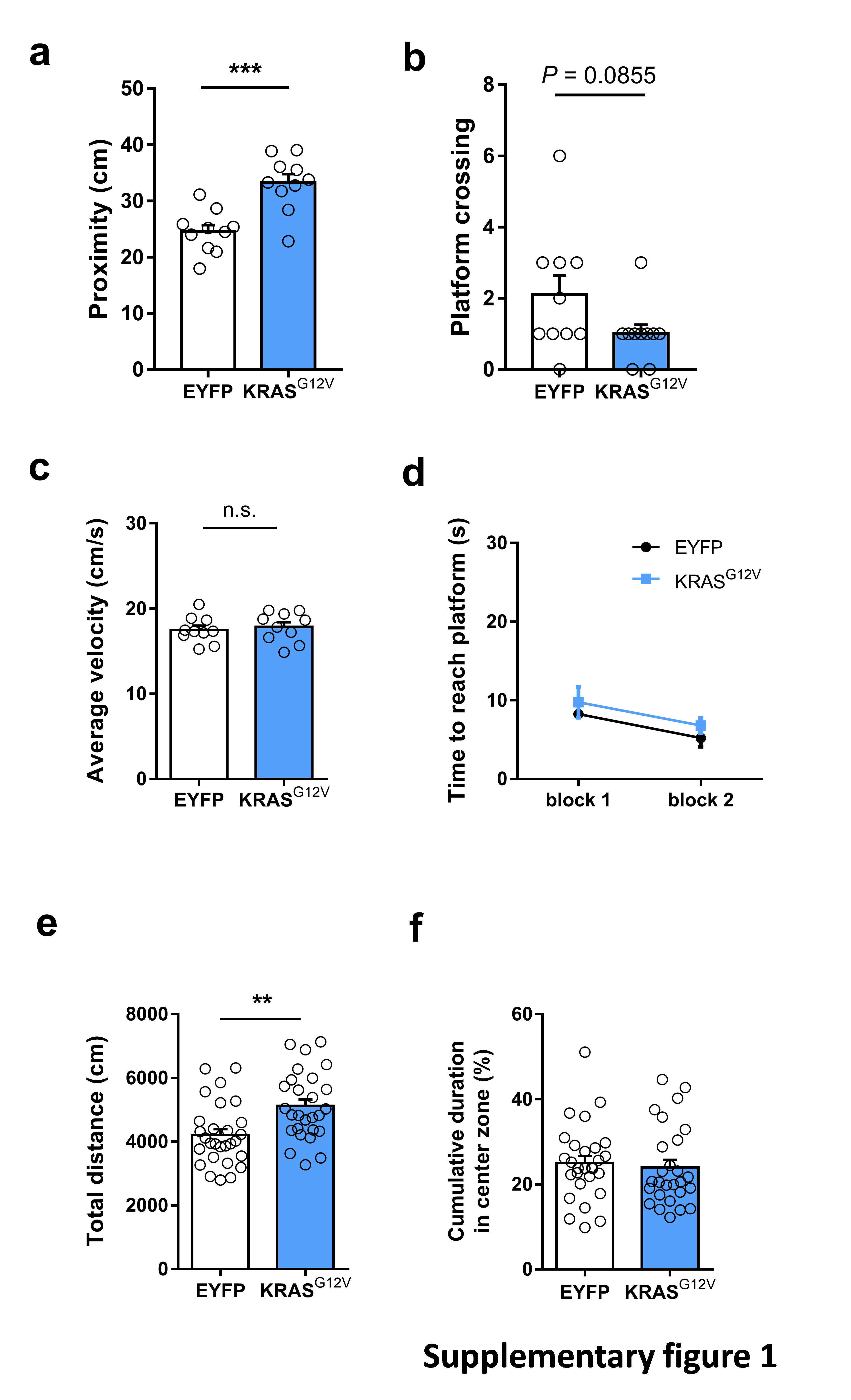

Supplement: Supplementary file 1 — Supplementary Figure 1. [file 41598_2020_74610_MOESM1_ESM.tif]

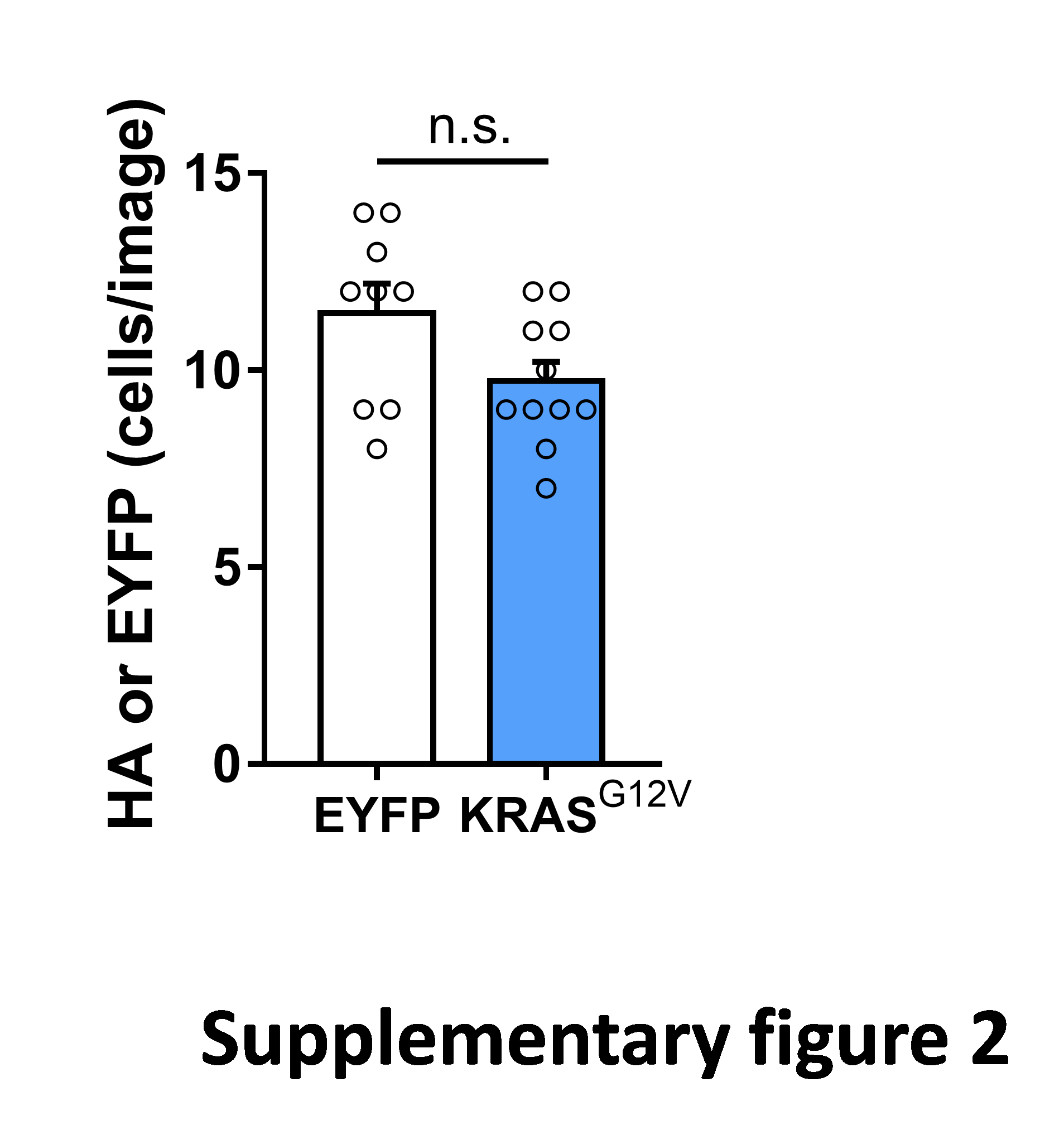

Supplement: Supplementary file 2 — Supplementary Figure 2. [file 41598_2020_74610_MOESM2_ESM.tif]

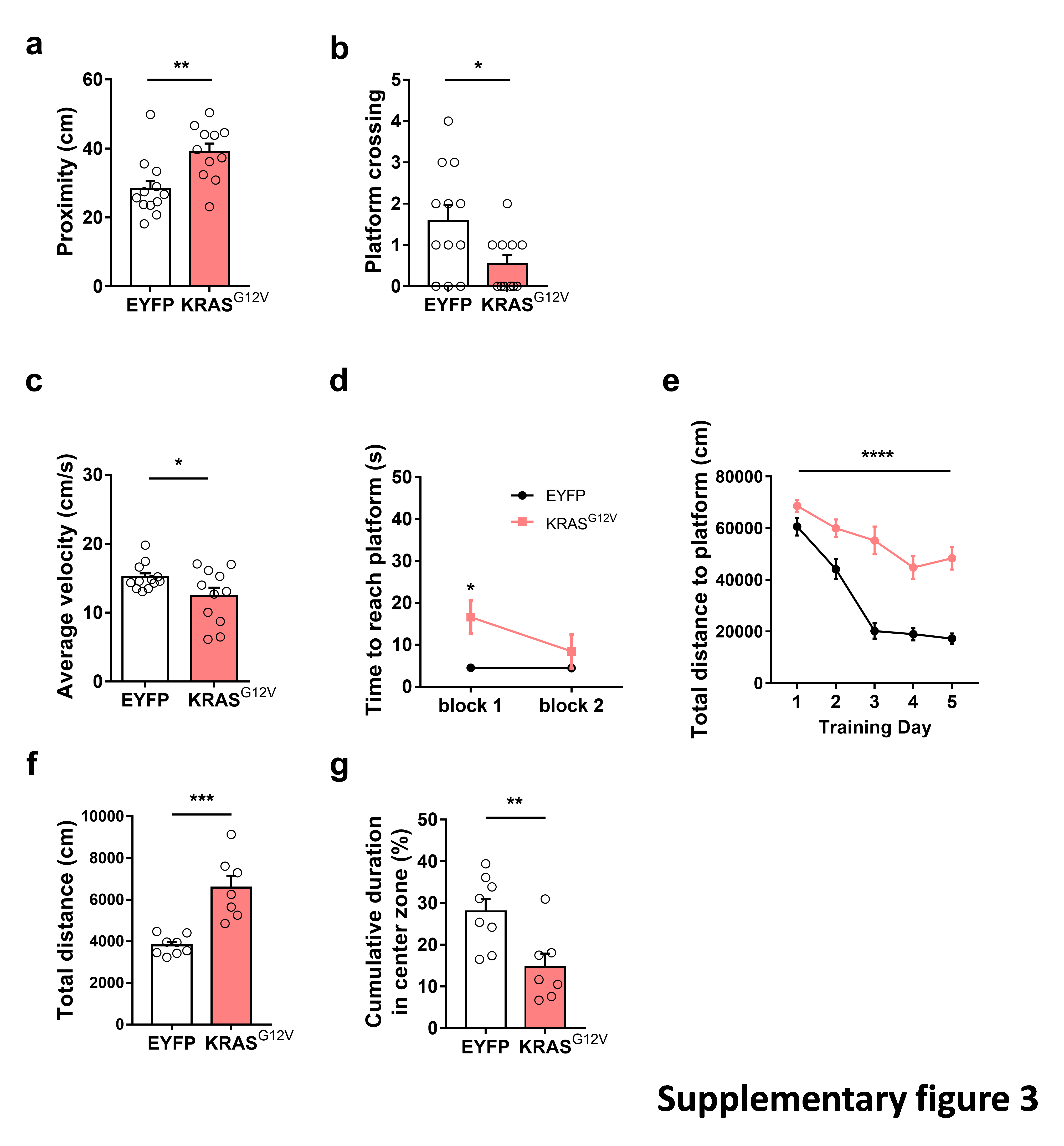

Supplement: Supplementary file 3 — Supplementary Figure 3. [file 41598_2020_74610_MOESM3_ESM.tif]

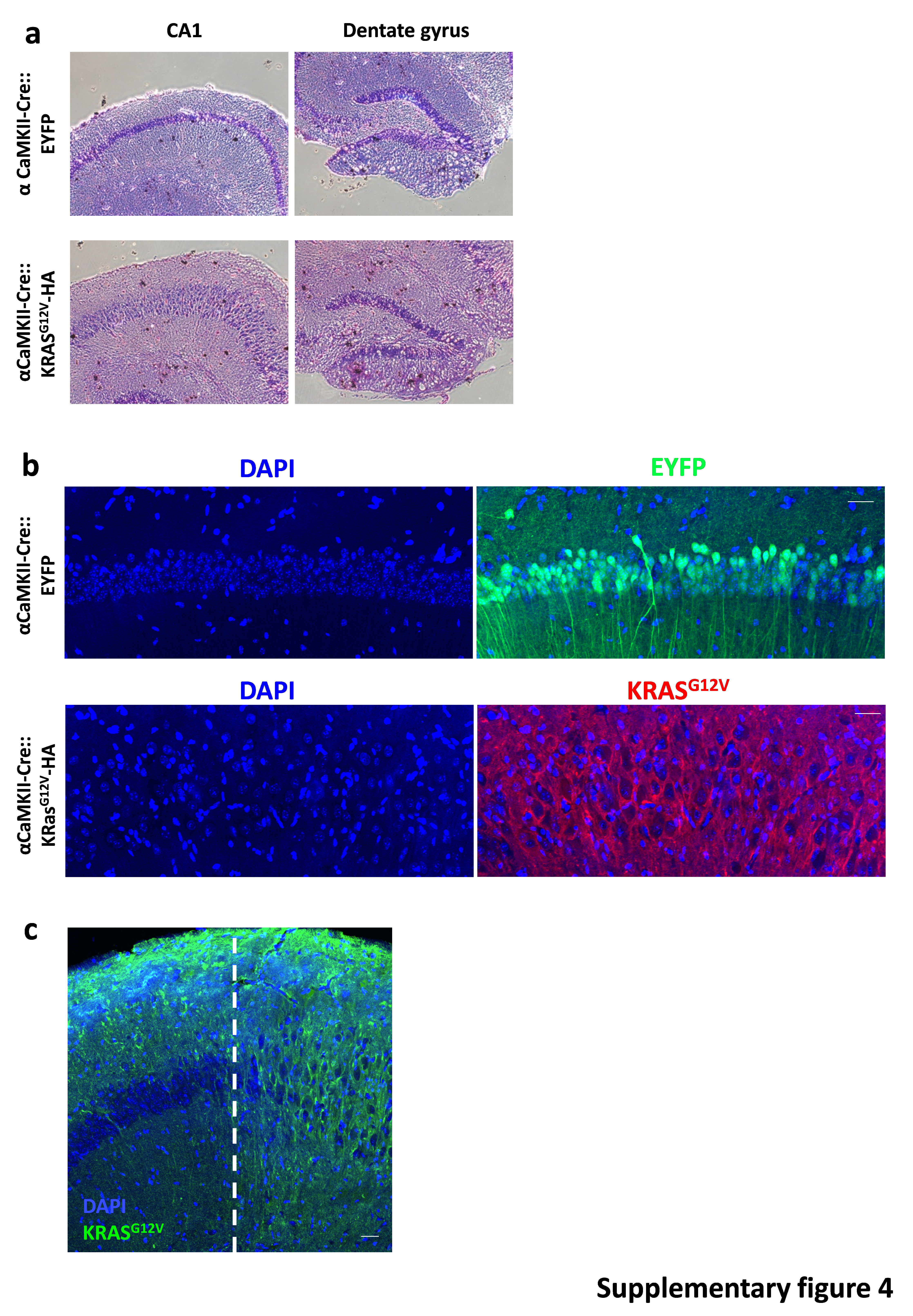

Supplement: Supplementary file 4 — Supplementary Figure 4. [file 41598_2020_74610_MOESM4_ESM.tif]

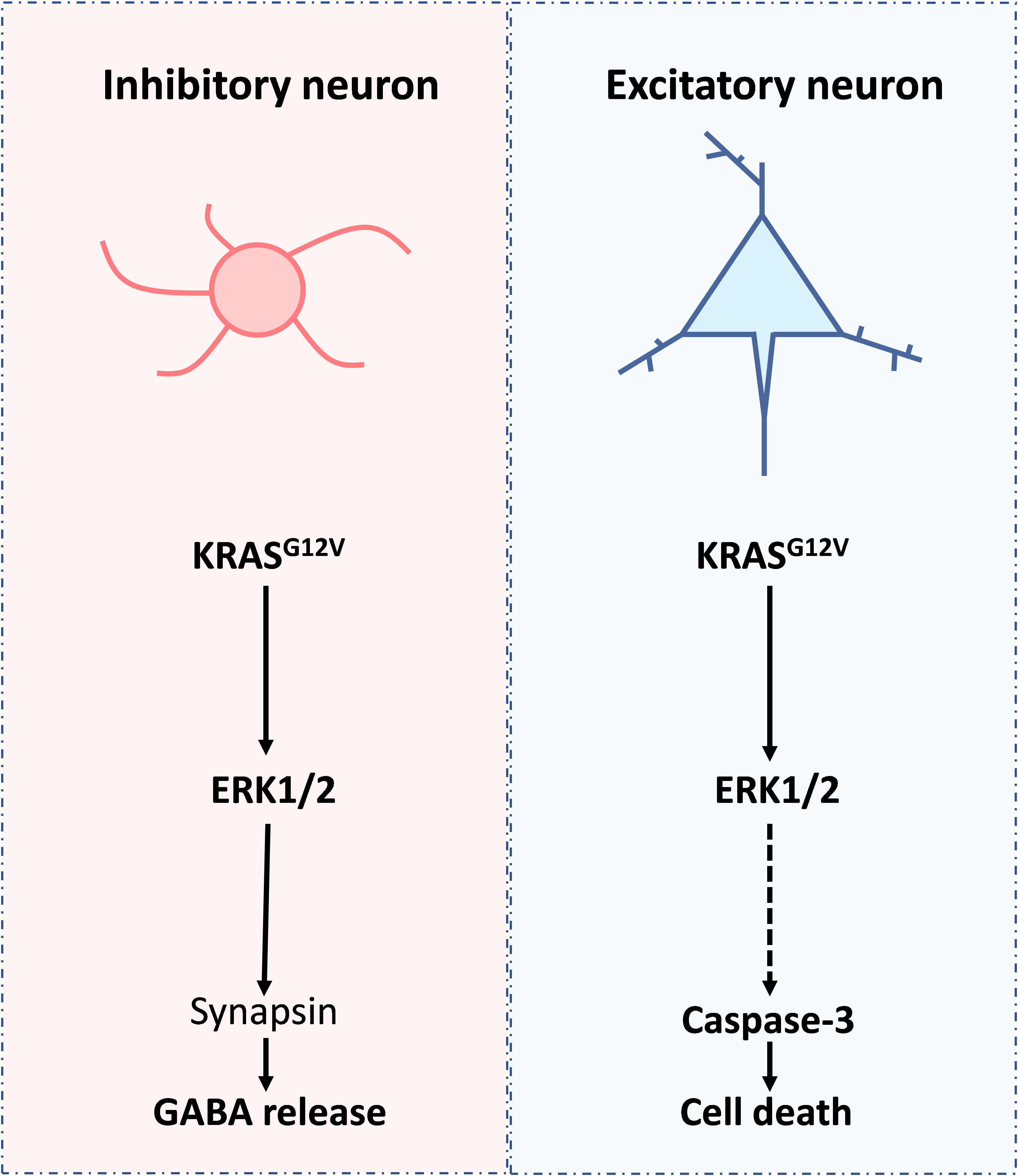

Supplement: Supplementary file 5 — Supplementary Figure 5. [file 41598_2020_74610_MOESM5_ESM.tif]
