# Supplementary material for: Neuron type-specific expression of a mutant KRAS impairs hippocampal-dependent learning and memory
Source: Sci Rep. 2020 Oct 20;10:17730. doi: 10.1038/s41598-020-74610-y (PMC7575532; doi:10.1038/s41598-020-74610-y)
Supplement: Supplementary file 6 — Supplementary Legend 6. [file 41598_2020_74610_MOESM6_ESM.docx]

**Supplementary Figure 1. Effects of ectopic expression of KRAS^G12V^ in inhibitory neurons on MWM and locomotive activity.** **(a)** Proximity to target platform (average distance to the platform's former location during the probe trial) was longer in vGAT-Cre::KRAS^G12V^ mice. Unpaired t-test, ****P* = 0.0003; **(b)** Number of platform crossing during the probe trial was tend to be decreased in vGAT-Cre::KRAS^G12V^ mice. Unpaired t-test, *P* = 0.0855; **(c)** Ectopic expression of KRAS^G12V^ in inhibitory neurons did not affect the swimming speed in the probe test. vGAT-Cre::EYFP, n = 10; vGAT-Cre::KRAS^G12V^, n = 10; unpaired t-test, *P* = 0.6358; **(d)** vGAT-Cre::EYFP and vGAT-Cre::KRAS^G12V^ mice showed comparable latencies to the target platform in the visible platform-version of water maze training. vGAT-Cre::EYFP, n = 10; vGAT-Cre::KRAS^G12V^, n = 10; Two-way repeated measure ANOVA, F_1, 18_ = 1.076, *P* = 0.3134; **(e)** vGAT-Cre::KRAS^G12V^ mice showed increased total distance moved in the open field test. vGAT-Cre::EYFP, n = 28; vGAT-Cre::KRAS^G12V^, n = 27; unpaired t-test, ***P* = 0.0017. **(f)** Both group of mice showed comparable cumulative duration in center zone in open field test. vGAT-Cre::EYFP, n = 28; vGAT-Cre::KRAS^G12V^, n = 27; unpaired t-test, *P* = 0.6795.

**Supplementary Figure 2. Total numbers of the KRAS^G12V^- or EYFP-expressing neurons in vGAT-Cre mice were not different.** The number of HA- or EYFP-positive neurons was comparable between vGAT-Cre::KRAS and vGAT-Cre::EYFP mice. vGAT-Cre::EYFP, n = 9 slices from 3 hippocampi; vGAT-Cre::KRAS^G12V^, n = 11 slices from 4 hippocampi. n.s., not significant. *P* = 0.0621. Data are expressed as the mean ± SEM.

**Supplementary Figure 3. Effects of ectopic expression of KRAS^G12V^ in excitatory neurons on MWM and locomotive activity.** **(a)** αCaMKII-Cre::KRAS^G12V^ mice swam significantly farther than αCaMKII-Cre::EYFP mice during probe trials. αCaMKII-Cre::EYFP, n = 12; αCaMKII-Cre::KRAS^G12V^, n = 11; unpaired t-test, ***P* = 0.0047; **(b)** αCaMKII-Cre::KRAS^G12V^ mice crossed the former platform position significantly less than αCaMKII-Cre::EYFP mice during probe trials. αCaMKII-Cre::EYFP, n = 12; αCaMKII-Cre::KRAS^G12V^, n = 11; unpaired t-test, **P* = 0.0290. **(c)** Swimming speed during the probe test was significantly reduced in αCaMKII-Cre::KRAS^G12V^ mice. αCaMKII-Cre::EYFP, n = 12; αCaMKII-Cre::KRAS^G12V^, n = 11; unpaired t-test, **P* = 0.0485; **(d)** αCaMKII-Cre:: KRAS^G12V^ mice showed longer latency to find the platform in the initial trials, but normally performed in the second trials. αCaMKII-Cre::EYFP, n = 7; αCaMKII-Cre::KRAS^G12V^, n = 6; Two-way repeated measure ANOVA, F_1, 11_ = 9.132, **P* = 0.0116. **(e)** Swimming distances to the platform during training trials were significantly longer in KRAS^G12V^ expressing mice compared to EYFP controls. αCaMKII-Cre::EYFP, n = 12; αCaMKII-Cre::KRAS^G12V^, n = 11; Two-way repeated measures ANOVA, F_1, 21_ = 47.4, *****P* < 0.0001. **(f)** αCaMKII-Cre::KRAS^G12V^ mice showed increased total distance moved in open field test. αCaMKII-Cre::EYFP, n = 8; αCaMKII-Cre::KRAS^G12V^, n = 7; unpaired t-test, ****P* = 0.0003. **(g)** αCaMKII-Cre::KRAS^G12V^ mice showed increased cumulative duration in center zone in open field test. αCaMKII-Cre::EYFP, n = 8; αCaMKII-Cre::KRAS^G12V^, n = 7; unpaired t-test, ***P* = 0.0096.

**Supplementary Figure 4. Ectopic KRAS^G12V^ expression in excitatory neuron results in morphological alterations in CA1.** **(a)** Nissl staining of hippocampal slices showed morphological alterations in the CA1 region of the KRAS^G12V^ expressing mice. **(b)** Representative immunohistochemistry images of hippocampal slices showing morphological alterations in the CA1 pyramidal cell layer of KRAS^G12V^ or EYFP expressing mice. Slices were immunostained for HA (green), and DAPI (blue) **(c)** Morphological abnormalities are clearly observed in the area where the virus expression is higher (right) compared to the region where the virus expression is relatively lower within the same hippocampus. Scale bar = 40 μm.

**Supplementary Figure 5. Simplified graphical summary for the impacts of KRAS hyperactivation on inhibitory and excitatory neurons.** In inhibitory neurons, KRAS^G12V^ activates ERK1/2, which phosphorylates synapsin I and subsequently enhances GABA release. In excitatory neurons, KRAS^G12V^ activates ERK1/2, which in turn promotes apoptosis by activating caspase-3.
